# How to Decide Approaches and Procedures for Early and Advanced Gastric Cancer?

**DOI:** 10.1155/2022/8324242

**Published:** 2022-04-04

**Authors:** Daisuke Izumi, Souya Nunobe

**Affiliations:** ^1^Department of Gastroenterological Surgery, Gastroenterological Center, Cancer Institute Hospital, Japanese Foundation for Cancer Research, Tokyo, Japan; ^2^Department of Gastroenterological Surgery, Graduate School of Medical Sciences, Kumamoto University, Kumamoto, Japan

## Abstract

In the 6th edition of the Japanese Gastric Cancer Treatment Guidelines, laparoscopic surgery is recommended as one of the standard treatments for cStage I. On the other hand, the recommendation of robot-assisted surgery for gastric cancer was also added, albeit not conclusively, to perform it for cStage I gastric cancer. Conversely, laparoscopic surgery for cStage II/III is not recommended, and several randomized controlled trials (RCTs) are being conducted in East Asia to expand the indication for advanced gastric cancer. Although laparoscopic surgery and robot-assisted surgery are now recommended in the Guidelines for Early-Stage Gastric Cancer, each institution should set its own criteria for indications according to its level of proficiency and try to provide high-quality treatment. For advanced gastric cancer, although there is no solid evidence for laparoscopic or robot-assisted surgery, the reality is that it is already being performed in facilities with ample experience. New evidence is expected to be reported in the future, based on which the recommendations may change.

## 1. Introduction

In the 6th edition of the Japanese Gastric Cancer Treatment Guidelines, laparoscopic surgery is recommended as one of the standard treatments for cStage I. On the other hand, the recommendation of robot-assisted surgery for gastric cancer was also added, albeit not conclusively, to perform it for cStage I gastric cancer [1]. Conversely, laparoscopic surgery for cStage II/III is not recommended, and several randomized controlled trials (RCTs) are being conducted in East Asia to expand the indication for advanced gastric cancer.

In this paper, we discuss the choice of approach for early and advanced gastric cancers based on the results of previous and ongoing clinical trials.

## 2. Laparoscopic Gastrectomy

**Figure 1:**
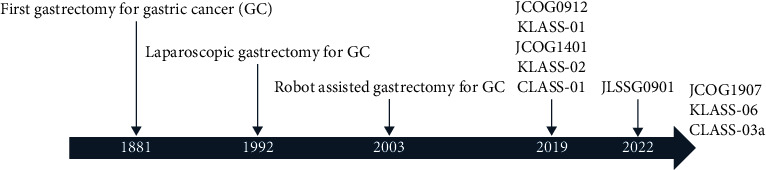
The historical timeline of gastrectomy.

Laparoscopic surgery for gastric cancer was first reported in 1994 [2], and almost 30 years have already passed since then (Figure 1). The development of techniques and the development and advancement of devices have greatly improved the safety and quality of surgery. Several large-scale prospective clinical trials and studies using big data have already proven its safety and oncological validity.

In the 5th edition of the Japanese Guidelines for the Treatment of Gastric Cancer, it was stated that “laparoscopic gastrectomy may be an option for routine practice in cStage I patients who are eligible for distal gastrectomy.” [3] However, in a subsequent preliminary guideline based on the results of a study by the Japan Clinical Oncology Group (JCOG), “laparoscopic distal gastrectomy (LADG), laparoscopic total gastrectomy (LATG), and laparoscopic proximal gastrectomy (LAPG) are recommended as one of the standard treatments for patients with cStage I gastric cancer.” However, the evidence for advanced gastric cancer is not sufficient, and the 6th edition of the same guideline states that “there is insufficient evidence to recommend LADG for gastric cancer of cStage II or higher [1].

### 2.1. cStage I

LADG was shown to be safe in JCOG0703, a phase II single-arm study with the primary endpoint of incidence of anastomotic leakage and pancreatic fistula [4]. In addition, JCOG0912 demonstrated noninferiority to open distal gastrectomy (ODG) in the primary endpoint of a 5-year recurrence-free survival (LADG 95.1% vs. ODG 94.0%) [5]. The same results were reported in an RCT (KLASS-01) conducted in Korea [6]. Based on these results, the 6th edition of the Japanese Gastric Cancer Treatment Guidelines states that “LADG is strongly recommended as one of the standard treatment options for cStage I gastric cancer.” [1] On the other hand, JCOG0912 states that LADG surgeons or leading assistants should be surgeons certified by the Japan Society for Endoscopic Surgery or surgeons certified by the group as having equivalent skills with experience of at least 30 cases of LADG. The safety of the procedure has been proven, and each institution should set its own criteria for indication according to the level of proficiency.

LATG and LAPG were shown to be safe in JCOG1401, a nonrandomized, single-arm study with the primary endpoint of the incidence of suture failure of esophageal jejunal anastomosis (grade 2–4 esophageal jejunal anastomotic suture failure: 2.5%) [7]. As for long-term results, it was considered to be acceptable to extrapolate the results of JCOG0912, but since no clear data were presented, the guideline only stated a weak recommendation to do so. Also in this study, the primary surgeon or leading assistant was strictly defined by the same criteria as in the JCOG0912 study.

To compare and validate the safety of LADG with ODG, a study using big data from the National Clinical Database (NCD) has also been conducted. In both the retrospective and prospective studies, the incidence of postoperative complications and mortality were similar to ODG; however, grade B pancreatic fistulas or higher were significantly more common in LADG [8]. NCDs have been studied in LATG/LAPG as well as in LADG, but a predominantly higher incidence of suture failure has been reported in LATG, especially in retrospective studies.

In addition to its safety and oncological relevance, laparoscopic gastrectomy should be actively performed for cStage I patients if the educating system is in place and based on proficiency of the surgeons, considering its less invasive nature and better esthetic appearance compared with open surgery.

### 2.2. cStage II/III

RCTs on LADG for advanced cancer have been conducted in East Asian countries. In Japan, JLSSG0901 by the Japan Laparoscopic Gastrectomy Study Group (JLSSG) is underway to investigate the safety and long-term results (follow-up completed in August, 2021). As for short-term results, they were reported in 2018 and showed that laparoscopic gastrectomy is safe [9]. Results on long-term outcomes will be reported in 2022. KLASS-02 in Korea and CLASS-01 in China demonstrated the noninferiority of LADG to ODG in terms of short-term results and 3-year recurrence-free survival [10, 11]. However, it has been emphasized that none of the trials proved the noninferiority of LADG from a statistical point of view, including the method of analysis and stability of results. Subcategory analysis also showed a trend toward poorer results in the LADG group in patients with serous invasion. In addition, the operative time and blood loss differed significantly from the results of JLSSG0901, suggesting that the details of the procedure may have differed; therefore, the final conclusion should await the results of JLSSG0901 performed in Japan.

As for RCTs on LATG for advanced cancer, KLASS-06 is ongoing in Korea, and JCOG1809, a single-arm study to evaluate the safety of laparoscopic spleen-sparing splenectomy, is ongoing in Japan. There was no significant difference in recurrence-free survival or overall survival between laparoscopic surgery and laparotomy in reports including total gastrectomy. However, only small-scale RCTs have been conducted, and further evidence is needed.

A multicenter study using big data in Japan also showed that the long-term outcome of laparoscopic surgery was not different from that of open surgery, but in an observational study of total gastrectomy using NCD, LATG resulted in significantly more anastomotic leakage than open gastrectomy [12].

Presently, laparoscopic surgery is indicated for cStage II/III patients only at centers with a lot of experience, and it is not yet the standard of care in Japan. In addition, the indications for serous invasion and cases requiring total resection should be considered carefully.

### 2.3. Postchemotherapy

Several prospective and retrospective studies on gastric cancer after chemotherapy have been conducted in Japan and China, but the sample size was not sufficient in any of them. A prospective RCT of 95 patients with advanced gastric cancer in a single center in China was conducted with 3-year recurrence-free survival as the primary endpoint. The short-term results showed that laparoscopic surgery was associated with fewer complications and a higher completion rate of postoperative adjuvant chemotherapy compared to laparotomies [13]. The long-term results are anticipated. In China, they are conducting a multicenter prospective RCT of laparoscopic surgery versus laparotomy after chemotherapy (CLASS-03a) and are currently recruiting patients.

The results so far are insufficient as evidence, and since surgery after chemotherapy has a high risk of complications, it is necessary to carefully select the approach method according to the indications of each institution and the proficiency of surgeons.

## 3. Robot-Assisted Gastrectomy

Robot-assisted gastrectomy for gastric cancer was first reported in 2003 (Figure 1) [14]; since then, many institutions have introduced robot-assisted gastrectomy.

Although laparoscopic gastrectomy for gastric cancer has already been widely spread, pancreas-related complications such as pancreatic fistula and intra-abdominal abscess are not uncommon problems. While various efforts have been made to prevent pancreatic fistula in laparoscopic surgery through preoperative image evaluation [15] and surgical manipulation [16], robot-assisted surgery is expected to enable the use of forceps with a three-dimensional field of view and a high degree of freedom with an antishake function, making it possible to safely and accurately perform procedures that are difficult to perform with conventional laparoscopic surgery.

### 3.1. cStage I

In a single-center phase II trial for cStage I gastric cancer, the primary endpoint was the incidence of intra-abdominal infectious complications of Clavien–Dindo classification (CD) grade II or higher, with an expected value of 4%, a threshold of 12%, and a one-sided confidence interval of 5%. The complication rate was 3.3% (4 cases), indicating the safety of robot-assisted gastrectomy [17]. In a multicenter prospective clinical trial, 330 patients with cStage I/II gastric cancer were enrolled, and the results showed that postoperative complications of CD grade IIIa or higher were reduced to 2.45%, less than half that seen in conventional laparoscopic surgery [18]. Conversely, an RCT conducted in Korea reported that there was no difference between robot-assisted and laparoscopic surgeries, with the respective values being 1.3% and 1.4% [19]. In Japan, an RCT (JCOG1907) is currently enrolling patients to evaluate the safety and superiority of robot-assisted gastrectomy over laparoscopic gastrectomy in cT1-2N0-2 gastric cancer with the primary endpoint of CD grade II or higher intra-abdominal infectious complications. As for long-term results, such as recurrence rate and survival, they are presumed to be similar to those of laparoscopic surgery, but evidence is insufficient.

The 6th edition of the Japanese Guidelines for the Treatment of Gastric Cancer states that although robot-assisted gastrectomy is as safe as laparoscopic gastrectomy and has the potential to reduce complications, the long-term results are unknown. The report weakly recommends the use of robot-assisted gastrectomy for cStage I patients, provided that it is performed by a certified physician who is proficient in this procedure, or under the guidance of a certified proctor [1].

### 3.2. cStage II/III

The safety of robot-assisted gastrectomy in cStage II has been demonstrated in a prospective multicenter clinical trial as described earlier. With regard to cStage III, the results of a multicenter prospective RCT in patients with cStage I–III disease showed that there was no difference in the incidence of intra-abdominal infectious complications, which was the primary endpoint. However, the incidence of all complications above CD grade II was significantly lower in the robot-assisted surgery. We are awaiting the results of the JLSSG0901 study, which examined the safety and long-term results of laparoscopic gastrectomy in advanced gastric cancer, and the results of JCOG1907, a randomized controlled study on robot-assisted gastrectomy versus laparoscopic gastrectomy in cT1-2N0-2 gastric cancer.

Until the evidence is established, robot-assisted gastrectomy for advanced cancer should be performed as a clinical trial by surgeons skilled in gastrectomy after considering the indications at each institution and fully explaining to patients the uncertainties of long-term and short-term outcomes.

## 4. Esophagogastric Junction Cancer

In the case of esophagogastric junction cancer, there is no consensus on the choice of surgical technique or approach in the Guidelines for Gastric Cancer Treatment, and the choice is currently left to the discretion of the surgeon or institution. The Japanese Gastric Cancer Society and the Japanese Esophageal Association conducted a prospective study on cT2-4 esophagogastric junction cancer and investigated the frequency of lymph node metastasis [20]. Although long-term results are not yet available, based on the results, it is considered reasonable to perform surgery using the trans-right thoracic approach for patients with esophageal invasion of more than 4 cm, the trans-esophageal hiatus approach for patients with esophageal invasion of 2 cm or less, and the appropriate approach in each institution for patients with invasion values that are within this range. There is no solid evidence on the choice of laparotomy, laparoscopic surgery, or robot-assisted surgery, and we await the publication of evidence in the future.

## 5. Conclusion

**Figure 2:**
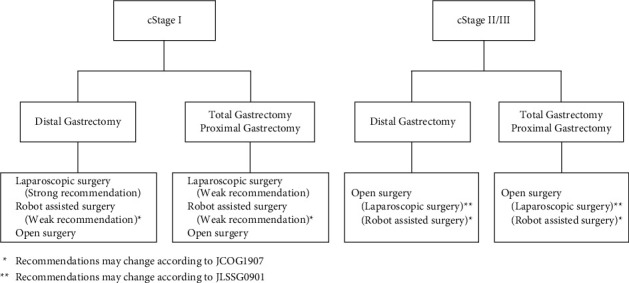
The selection of the current evidence-based surgical approach for gastrectomy for gastric cancer.

The selection of the current evidence-based surgical approach for gastrectomy for gastric cancer was described (Figure 2). Significant advancement of medical technologies and robust clinical trials lead to dramatic change in choosing approach for gastrectomy in the last decade (Table 1). Although laparoscopic surgery and robot-assisted surgery are now recommended in the Guidelines for the Treatment of Gastric Cancer for early-stage gastric cancer, each institution should set its own criteria for indications according to its level of proficiency and try to provide high-quality treatment. As considering the criteria of operator, an objective and evidence-based criteria should be needed. For advanced gastric cancer, although there is no solid evidence for laparoscopic or robot-assisted surgery, the reality is that it is already being performed in facilities with ample experience. New evidence is expected to be reported in the future, based on which the recommendations may change.

## Figures and Tables

**Table 1:** Multicenter prospective trials for minimal-invasive gastric cancer surgery.

Trials	Main outcomes	Authors	Year
JCOG0703	Safety of LADG.	Kurokawa et al.	2008
JCOG0912	Noninferiority of LADG to ODG regarding survival outcomes for early gastric cancer.	Katai et al.	2019
KLASS-01	Noninferiority of LADG to ODG regarding survival outcomes for early gastric cancer.	Kim et al.	2019
JCOG1401	Safety of LATG and LAPG.	Katai et al.	2019
KLASS-02	Noninferiority of LADG to ODG regarding safety and survival outcomes for advanced gastric cancer.	Lee and hyung et al.	2019 and 2020
CLASS-01	Noninferiority of LADG to ODG regarding safety and survival outcomes for advanced gastric cancer.	Yu and huang et al.	2019 and 2022
JLSSG0901	Noninferiority of LADG to ODG regarding safety and survival outcomes for advanced gastric cancer.	Ongoing	
KLASS-06	Noninferiority of LATG to OTG regarding safety and survival outcomes for advanced gastric cancer		
JCOG1907	Superiority of RAG to LAG regarding safety and survival outcomes for T1-2N0-2 gastric cancer.	Ongoing	
CLASS-03a	Safety of LAG after neoadjuvant chemotherapy.	Ongoing	

LADG, laparoscopic distal gastrectomy. ODG, open distal gastrectomy. LATG, laparoscopic total gastrectomy. LAPG, laparoscopic proximal gastrectomy. OTG, open total gastrectomy. RAG, robot-assisted gastrectomy. LAG, laparoscopic gastrectomy.

## Data Availability

The data supporting this REVIEW are from previously reported studies and datasets, which have been cited.
